# Analysis of Risk Factors for Anorectal Malformations Combined with Tethered Cord Syndrome and the Impact of Untethering Surgery on Anorectal Function in Related Children: Preliminary Results from a Single-Center Study

**DOI:** 10.3390/children11121504

**Published:** 2024-12-10

**Authors:** Tao-Tao Zhang, Yan-Bing Huang, Yu-Yan He, Fan Chen, Jian-Bin Ying, Shou-Qing Sun, Qing-Shuang Zhao, Jun-Jie Jing

**Affiliations:** 1 Department of Neurosurgery, Fujian Children’s Hospital (Fujian Branch of Shanghai Children’s Medical Center), College of Clinical Medicine for Obstetrics & Gynecology and Pediatrics, Fujian Medical University, Fuzhou 350000, China; zhangtaotao@fjsetyy.com (T.-T.Z.); heyuyan@fjsetyy.com (Y.-Y.H.); chenfan@fjsetyy.com (F.C.); yingjianbin@fjsetyy.com (J.-B.Y.); sunshouqing@scmc.com.cn (S.-Q.S.); zhaoqingshuang@fjsetyy.com (Q.-S.Z.); 2 Department of Pediatric Surgery, Fujian Children’s Hospital (Fujian Branch of Shanghai Children’s Medical Center), College of Clinical Medicine for Obstetrics & Gynecology and Pediatrics, Fujian Medical University, Fuzhou 350000, China; huangyanbing@fjsetyy.com; 3 Department of Neurosurgery, Shanghai Children’s Medical Center Affiliated to Medical School of Shanghai Jiaotong University, Shanghai 200120, China

**Keywords:** anorectal malformations (ARMs), tethered cord syndrome (TCS), risk factors, anorectal function, children

## Abstract

Background: Anorectal malformations (ARMs) are often associated with tethered cord syndrome (TCS). This study focused on children with ARM to explore the risk factors for the co-occurrence of TCS and to investigate the impact of untethering surgery on anorectal function among these children. Methods: A retrospective analysis was conducted on 130 children with ARM treated at Fujian Provincial Children’s Hospital (Fujian Hospital of Shanghai Children’s Medical Center) from May 2021 to January 2024. A total of 114 children were included in the study on the basis of the inclusion and exclusion criteria. The patients were divided into two groups according to the presence of TCS: the ARM group (n = 83) and the ARM+TCS group (n = 31). to explore the risk factors for the co-occurrence of ARM and TCS. All children diagnosed with TCS underwent untethering surgery regardless of symptoms. Anorectal function was compared between the ARM and ARM+TCS groups. Results: In the multivariate analysis, intermediate or high-type ARM increased the risk of children with ARM having TCS, with an OR of 3.572, 95% CI from 1.355 to 9.418, and *p* = 0.010. Additionally, the presence of other malformations also increased the risk of children with ARM having TCS (*p* = 0.026). When the ARM+TCS group was compared with the ARM group, children with low-type ARM in the ARM+TCS group exhibited a significant improvement in constipation following untethering surgery (*p* = 0.043). However, when children with intermediate or high-type ARM were compared, the anorectal function of the children in both groups was comparable. Conclusions: Intermediate or high-type ARM and the presence of other malformations are risk factors for the co-occurrence of TCS in children with ARM. In children with low-type ARM, those with TCS and ARM showed significant improvement in constipation after untethering surgery compared with those with ARM without TCS. We recommend that children with relevant conditions actively undergo untethering surgery.

## 1. Introduction

Anorectal malformations (ARMs) are a type of congenital disease involving abnormalities of the anus and rectum, with an incidence of 1 in 5000 to 1 in 2000. The occurrence of this malformation may be related to genetic factors and environmental factors during pregnancy (such as viral infections, chemical substances, nutritional status, etc.). In addition, the mother’s age, socioeconomic status, and lifestyle may also be associated with the risk of ARM occurrence [1]. ARMs are often associated with other malformations, with an incidence rate of 28% to 72%, commonly including urogenital, cardiac, and spinal limb malformations [2,3,4]. Between 20% and 60% of ARMs are associated with TCS [5,6]. TCSs are a group of neurological dysfunctions and deformities caused by congenital factors. Untethering surgery can often halt clinical deterioration and may reverse it [7,8]. TCS is also a congenital disease, and by linking these two conditions, we can see that they both involve abnormalities in embryonic development. The ARM and TCS may share certain risk factors, which are currently unknown.

With respect to the treatment of ARM, surgery is the only effective method. However, due to the complex pathological types of ARMs, their postoperative efficacy is often uncertain. After anoplasty, issues such as anal stenosis, rectal mucosal eversion, and anal incontinence may arise, leading to defecation dysfunction [9]. TCS occurs when the spinal cord is abnormally tethered by tissue, limiting its movement. This condition can also lead to bowel control disorders, including constipation or incontinence [8]. When children have both ARM and TCS, anorectal function after untethering surgery has not been effectively evaluated, and there is controversy over whether to perform untethering surgery [10,11].

This study retrospectively reviewed the medical history, imaging data, and follow-up information of children with ARM treated at Fujian Provincial Children’s Hospital, aiming to explore the risk factors for the comorbidity of TCS. By comparing the postoperative defecation symptoms of the relevant children, this study explored whether untethering surgery could improve the anorectal function of children with ARM combined with TCS, which also provides a basis for the clinical management of children with ARM and TCS.

## 2. Materials and Methods

### 2.1. Patients

**Figure 1:**
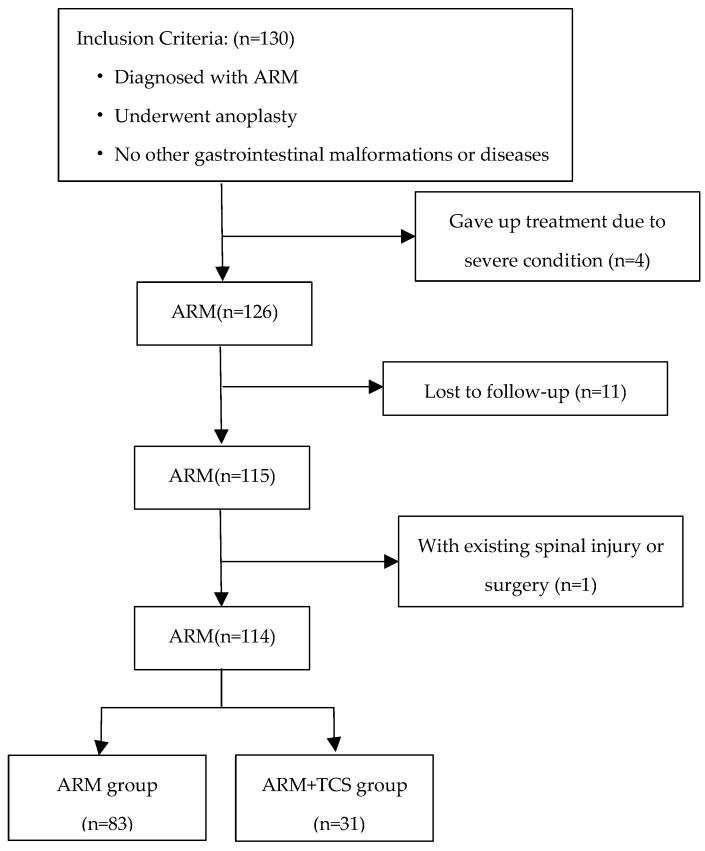
Inclusion and exclusion criteria for studies on ARM. ARM, Anorectal malformation; TCS, tethered cord syndrome.

This study was approved by the Medical Ethics Committee of Fujian Provincial Children’s Hospital (No. 2024ETKLRK07005), and all legal guardians of the children signed the informed consent form for this clinical study. This retrospective analysis focused on 130 children with ARM admitted to Fujian Provincial Children’s Hospital (Fujian Hospital of Shanghai Children’s Medical Center) from May 2021 to January 2024. The inclusion criteria for case selection were as follows: ① age is less than or equal to 16 years old; ② all the children were followed up at least half a year after anoplasty and 3 months after untethering surgery; ③ complete medical history and lumbar sacral MRI examination; ④ all children underwent anoplasty; and ⑤ absence of gastrointestinal structural abnormalities or diseases other than ARM. The exclusion criteria were as follows: ① children who abandoned treatment due to severe illness; ② exclusion of children with a history of spinal cord injury or surgery; ③ were lost to follow-up; and ④ presence of other neuromuscular diseases. A total of 114 children met the inclusion and exclusion criteria, including 83 children in the ARM group and 31 children in the ARM+TCS group (Figure 1).

### 2.2. Data Collection

All the data were sourced from the hospital’s big data platform, WeChat platform, and postoperative follow-up. The following information was extracted: (1) Demographic characteristics, including sex, age at surgery, gestational age, and number of pregnancies. (2) Disease information encompassing wing spread classification, Krickenberger classification, TCS type, and the presence of other malformations. (3) Maternal and paternal reproductive history, including parental age at conception, mode of conception, abnormal pregnancy history, history of maternal diseases, labor, active smoking or passive smoking during pregnancy, folic acid supplementation, maternal infection in early pregnancy, and parental education level. All the children were followed up at least half a year after anoplasty and 3 months after untethering surgery, and bowel movement symptoms, which included constipation, soiling, and voluntary bowel movement, were assessed via the Krickenbeck clinical scoring system. Voluntary bowel movement ability was only recorded if the child met all three conditions: feeling of urge, capacity to verbalize, and ability to hold stools.

All pediatric patients underwent lumbosacral 3.0T magnetic resonance imaging (MRI) scans with adequate sedation prior to examination. The images collected included axial, sagittal, and coronal T1-weighted imaging (T1WI) and T2-weighted imaging (T2WI) of the lumbosacral region. The assessments and reports were made by senior pediatric neuroradiologists to determine the presence of TCS. The types of TCS included: spinal lipomas (SLs), spinal diastematomyelia, meningocele, myelomeningocele, lipomyelomeningocele, meningomyelocele, syringomyelia, low conus position (below the T2 level and over 3 months of age), and a thickened filum terminale (greater than 2 mm).

#### 2.2.1. Research Design

Patients were divided into two groups according to the presence or absence of TCS in patients with ARM: the ARM group and the ARM+TCS group. The demographic characteristics, disease information, and maternal pregnancy history of the patients in both groups were collected. Univariate and multivariate analyses were conducted to explore the risk factors for ARM combined with TCS. Although the regularity of ARM combined with TCS has been identified, the impact of untethering surgery on the anorectal function of affected children is unknown. Therefore, we assessed anorectal function in ARM patients with TCS after untethering surgery and compared it with that of ARM patients without TCS.

#### 2.2.2. Statistical Analysis

Statistical analysis was performed via SPSS software version 24.0. Categorical variables are represented by frequencies and rates, intergroup comparisons were made using the chi-square test, and ordinal variables were analyzed with the rank sum test. Logistic regression analysis was employed to analyze the risk factors for ARMs combined with TCS. A *p* value of less than 0.05 (two-tailed) was considered statistically significant.

## 3. Results

### 3.1. Risk Factors for ARMs Associated with TCS

**Figure 2:**
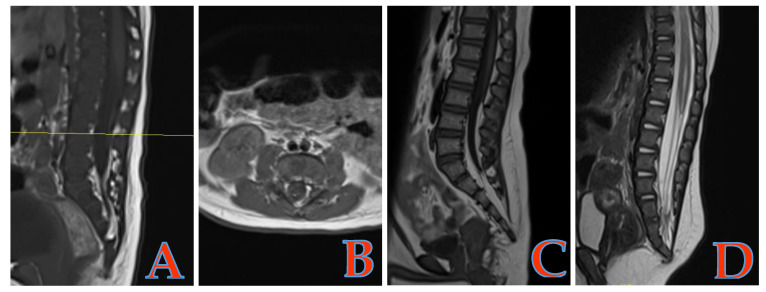
Type IV SL: The conus medullaris is in a normal position, and there is partial fatty degeneration of the filum terminale, with punctate high signals appearing on the axial T1WI. (**A**,**B**) Type III SL: The lipoma is located below the conus medullaris, merging with the subcutaneous fat through the sacral hiatus, and the entire length of the filum terminale has undergone fatty degeneration. (**C**) The conus medullaris has experienced dilation of the central canal. (**D**) SL, spinal lipomas; T1WI, T1-weighted images.

Among 114 children with ARM, 31 cases (27.2%) were associated with TCS, of which 30 cases were SL (4 cases were type III, 26 cases were type IV), and 1 case was syringomyelia (Figure 2). Seventy-five patients (65.79%) had other malformations (including congenital heart disease, urogenital system malformations, skeletal system malformations, Down syndrome, VATER association, etc.), with 47 patients having one additional malformation and 28 patients having two or more additional malformations.

The results of the univariate analysis affecting the combination of ARM and TCS are as follows (Table 1). Intermediate or high-type ARM and the presence of other malformations are associated with an increased risk of ARM combined with TCS. However, there was no statistically significant difference between the two groups in terms of age, parental reproductive age, maternal nonadherence to folic acid supplementation, history of infection in early pregnancy, abnormal pregnancy history, active smoking or passive smoking history, maternal education level, multiple pregnancy, conception method, labor, gestational age, or history of maternal diseases.

All variables were included in the multivariate analysis. Intermediate or high-type ARM also increased the risk of ARM children having TCS, with an OR of 3.572, 95% CI from 1.355 to 9.418, and a *p* value of 0.010. Additionally, the presence of other malformations also increases the risk of ARM children having TCS, with a *p* value of 0.026 (Table 2).

### 3.2. Anorectal Function with an ARM Combined with TCS After Untethering Surgery

Patients with low-type ARM underwent one-stage anoplasty, whereas those with intermediate or high-type ARM underwent a multistage treatment protocol (stage one involves a colostomy of the transverse colon, stage two involves anoplasty, and stage three involves closure of the colostomy). All patients in the ARM+TSC group underwent tethered cord release following surgical treatment of the ARM, with a median age of 29.7 months (ranging from 2 to 120 months) at the time of surgery. One patient with cerebrospinal fluid leakage from the incision site was cured with a pressure dressing. We did not assess preoperative anorectal function, as most patients were too young. In terms of constipation, children in the ARM+TCS group demonstrated significantly better anorectal function than those in the ARM group with low-type ARM (Table 3). When anorectal function was compared between the two groups of children with intermediate or high-type ARM, there was a decrease in the proportion of children with the ability to defecate independently, without constipation or fecal soiling. No statistically significant difference was observed between the two groups (Table 4).

## 4. Discussion

ARM is caused by abnormal development of the anorectal region during the embryonic period, and while the exact etiology is currently unclear; it is currently believed to be the result of the combined action of genetic and environmental factors [12]. ARM often coexists with multiple malformations, including TCS. Based on the results of this study, the author believes that the risk factors for the coexistence of ARM with TCS in children have the following characteristics:

1. The proportion of children in the ARM+TCS group who took folic acid promptly was lower than that in the ARM group, although there was no significant difference between the two groups. Folic acid is an essential substance for the development of the nervous system during the embryonic stage. Deficiency can affect the fetal nervous system, leading to the occurrence of TCS [13]. Many pregnant women only discover their pregnancy after a delayed menstrual period, at which time they are already 5–6 weeks postovulation, and the neural tube has already closed, making it too late to begin taking folic acid [14].

2. The prevalence of TCS is greater in children with intermediate or high-type ARM, and the occurrence of TCS is not related to the Krickenbeck classification, which is consistent with the literature [15,16]. The probability of TCS in children with intermediate or high-type ARM is 3.572 times greater than that of children with low-type ARM. When children with ARM are found to have multiple system malformations, the risk of TCS is greater, and screening for TCS should receive more attention at this time.

3. This study revealed that the incidence rate of TCS in children with ARM is approximately 27.2%, which is close to the results reported in domestic and international literature (20% to 60%), but it is much higher than the incidence rate of TCS in newborns (2.5/10,000 to 2.7/1000) [17]. Given the high detection rate of TCS, it is recommended that all children with ARM undergo lumbosacral MRI before surgery. In addition to the timely identification of children with TCS, MRI has high accuracy in the preoperative typing of ARM, assessment of sphincter development, and accurate location of fistulas [5,18,19]. Some scholars believe that sacrococcygeal ultrasound has high specificity for diagnosing TCS, but sacrococcygeal ultrasound cannot accurately detect filar lipomatosis, and its sensitivity is relatively low [20]. Furthermore, sacrococcygeal ultrasound must be conducted before ossification of the posterior arch of the lumbar vertebrae, a time window that is usually only three months.

4. In this study, out of 31 cases of TCS, 30 cases were classified as spinal lipomas. All of these SLs are Type III or Type IV [21]. During embryonic development, the formation of the primary neural tube occurs between approximately 17 and 26 days postovulation, and the formation of the secondary neural tube occurs between 26 and 49 days postovulation [22,23]. Failure of the cloacal septum to divide leads to ARM, which occurs between 5 and 8 weeks postovulation and overlaps with the late stage of secondary neural tube formation. Therefore, when an ARM is associated with a TCS, the type of TCS is typically Type III or Type IV SL, which occurs after primary neural tube formation.

As surgical techniques and skills have progressed and the perioperative management of children with ARMs has increased, the complications associated with ARMs have seen significant improvement. However, postoperative constipation or fecal incontinence remain the most common complications affecting the quality of life of children with these malformations [24]. Numerous studies have reported on anorectal function in children with ARM who also have TCS after untethering surgery, with varying results [25,26]. We assessed defecation symptoms in children with ARM who underwent TCS following untethering surgery and compared them with those of ARM children without TCS. We discovered that in children with low-type ARM, those in the ARM+TCS group exhibited a significant improvement in constipation symptoms after untethering surgery compared with the children in the ARM group. When anorectal function was compared between the two groups of children with intermediate or high-type ARM, no statistically significant difference was observed. The act of defecation is a complex physiological process that can be influenced by colonic motility, anorectal morphology, pelvic floor function, and sphincter function. Animal experiments also suggest that in severe ARM, the neural pathways that control defecation may not fully develop during the embryonic stage, and untethering surgery performed subsequently may not improve anorectal function [27]. In contrast, children with low-type ARM have a significantly lower chance of congenital defects and surgical complications. Constipation is the most common early symptom of TCS, and in children with low-type ARM and TCS, untethering surgery can markedly improve symptoms of constipation. In the future, we will also conduct more clinical and basic experiments to explore the underlying mechanisms. We strongly recommend that children with low-type ARM who present with constipation as the primary symptom undergo untethering surgery upon diagnosis of TCS. This procedure can improve constipation symptom. For other children with ARM and TCS, we recommend undergoing untethering surgery as well, given that delayed intervention poses risks. New neurological deficits, such as urinary dysfunction, lower limb weakness, and foot deformities, can occur at any time while TCS is present. Furthermore, the use of neurophysiological monitoring can increase the safety of untethering surgery [28].

This study also has several limitations: 1. As a retrospective study, during the outpatient follow-up or WeChat follow-up process, many risk factors require the parents of the children to recall, and some factors are indeed difficult to remember accurately, leading to biases in the accuracy of some data; 2. Some risks involve sensitive social information, such as the mother’s smoking behavior, whether active or passive, and the real situation is likely to be more severe than what is reflected in the study; 3. Another limitation is that this study is a single-center study, and there are insufficient sample sizes for some groups, which may lead to biases in the research results; and 4. Due to the young age of many children, they are unable to complete anorectal manometry, resulting in a lack of objective examination results for anorectal function assessment.

In summary, having intermediate or high-type ARM and having other associated malformations are risk factors for the co-occurrence of TCS in children with ARM. Children with low-type ARM who also have TCS tend to have better anorectal function after untethering surgery than those with low-type ARM who do not have TCS. However, among children with intermediate or high-type ARM, anorectal function is comparable between the two groups.

## Figures and Tables

**Table 1:** Univariate analysis of risk factors for ARMs combined with TCS.

Variables	ARM Group (n = 83)	ARM+TCS Group (n = 31)	OR	95% CI	*p*
Sex					
Female	32 (38.55)	12 (38.71)	0.332	(0.072–1.540)	0.159
Male	51 (61.45)	19 (61.29)			
Maternal age at childbirth (years)					
<35	62 (74.70)	26 (83.87)	1.949	(0.155–7.334)	0.545
>=35	21 (25.30)	5 (16.13)			
Paternal age at childbirth (years)					
<35	51 (61.45)	24 (77.42)	1.065	(0.155–7.334)	0.949
>=35	32 (38.55)	7 (22.58)			
Education level of mother					
Elementary school	6 (7.23)	3 (9.68)	Ref.	Ref.	0.722
Junior high school	24 (28.92)	9 (29.03)	0.402	(0.059–2.741)	0.352
High school	21 (25.30)	5 (16.13)	0.616	(0.074–5.117)	0.654
University or Above	32 (38.55)	14 (45.16)	0.818	(0.117–5.725)	0.839
Active smoking or passive smoking					
Yes	22 (26.51)	11 (35.48)	2.431	(0.737–8.021)	0.145
No	61 (73.49)	20 (64.52)			
Taking folic acid on time					
Yes	34 (40.96)	6 (19.35)	2.640	(0.679–10.268)	0.161
No	49 (59.04)	25 (80.65)			
Abnormal pregnancy history					
Yes	23 (27.71)	6 (19.35)	0.627	(0.181–2.175)	0.462
No	60 (72.29)	25 (80.65)			
Mode of conception					
Natural	77 (92.77)	28 (90.32)	0.380	(0.047–5.117)	0.365
Assisted	6 (7.23)	3 (9.68)			
Multiple pregnancy					
Yes	2 (2.41)	1 (3.23)	1.317	(0.051–34.285)	0.869
No	81 (97.59)	30 (96.77)			
Infection in early pregnancy					
Yes	17 (20.48)	12 (38.71)	0.379	(0.102–1.412)	0.148
No	66 (79.52)	19 (61.29)			
Maternal diseases					
Yes	15 (18.07)	4 (12.90)	0.954	(0.174–5.226)	0.957
No	68 (81.93)	27 (87.10)			
Gestational age (weeks)					
<37	17 (20.48)	4 (12.90)	0.378	(0.076–1.875)	0.234
>=37	66 (79.52)	27 (87.10)			
Labor					
Vaginal birth	50 (60.24)	19 (61.29)	0.726	(0.213–2.476)	0.609
Cesarean section	33 (39.76)	12 (38.71)			
Wingspread classification					
Low	66 (79.52)	14 (45.16)	0.117	(0.029–0.468)	0.002
Intermediate or high	17 (20.48)	17 (54.84)			
Krickenberger classification					
Rectourethral fistula	13 (15.66)	7 (22.58)	Ref.	Ref.	0.966
Rectovesical fistula	3 (3.61)	3 (9.68)			0.998
Rectovaginal fistula	2 (2.41)	1 (3.23)			0.998
Anal stenosis	9 (10.84)	3 (9.68)			0.998
Perineal fistula	30 (36.14)	7 (22.58)			0.999
Rectourethrovaginal fistula	25 (30.12)	8 (25.81)			0.999
Rectal atresia	1 (1.20)	2 (6.45)			0.999
Presence of other malformations					
No	36 (43.37)	3 (9.68)	Ref.	Ref.	0.014
One type	32 (38.55)	15 (48.39)	6.528	(1.571–27.130)	0.010
Two types or more	15 (18.07)	13 (41.94)	7.701	(1.686–35.171)	0.008

The values are presented as numbers and percentages (%). TCS, tethered cord syndrome; ARM, anorectal malformation; OR, odds ratio; CI, confidence interval.

**Table 2:** Multivariate analysis of risk factors for ARMs combined with TCS.

Variables	B	SE	OR	95% CI	*p*
Wingspread classification	1.273	0.495	3.572	(1.355–9.418)	0.010
Presence of other malformations					
No	Ref.	Ref.	Ref.	Ref.	0.026
One type	1.335	0.615	3.801	(1.139–12.688)	0.030
Two types or more	1.743	0.669	5.716	(1.541–21.210)	0.009

SE, standard error; OR, odds ratio; CI, confidence interval.

**Table 3:** Comparison of defecation symptoms between the two groups with low ARM.

Variables	ARM Group (n = 65)	ARM+TCS Group (n = 14)	χ^2^/Z	*p*
Soiling				
No	35 (53.85)	9 (64.28)	−0.589	0.556
Grade 1 once or twice per week	12 (18.46)	2 (14.29)		
Grade 2 Every day, no social problem	10 (15.38)	1 (7.14)		
Grade 3 Constant, social problem	8 (12.31)	2 (14.29)		
Constipation				
No	21 (32.31)	9 (64.28)	−2.020	0.043
Grade 1 Manageable by changes in diet	19 (29.23)	2 (14.29)		
Grade 2 Requires laxatives	11 (16.92)	2 (14.29)		
Grade 3 Resistant to diet and laxatives	14 (21.54)	1 (7.14)		
Voluntary bowel movements				
No	22 (33.85)	3 (21.43)	0.821	0.365
Feeling of urge, Capacity to verbalize, Hold defecation	43 (66.15)	11 (78.57)		

The values are presented as numbers and percentages (%). ARM, anorectal malformation; TCS, tethered cord syndrome.

**Table 4:** Comparison of defecation symptoms between the two groups with intermediate or high-type ARM.

Variables	ARM Group (n = 18)	ARM+TCS Group (n = 17)	χ^2^/Z	*p*
Soiling				
No	8 (44.44)	6 (35.29)	−0.434	0.665
Grade 1 once or twice per week	5 (27.78)	5 (29.41)		
Grade 2 Every day, no social problem	2 (11.11)	4 (23.53)		
Grade 3 Constant, social problem	3 (16.67)	2 (11.77)		
Constipation				
No	5 (27.78)	7 (41.18)	−0.326	0.744
Grade 1 Manageable by changes in diet	7 (38.88)	3 (17.65)		
Grade 2 Requires laxatives	3 (16.67)	5 (29.41)		
Grade 3 Resistant to diet and laxatives	3 (16.67)	2 (11.76)		
Voluntary bowel movements				
No	12 (66.67)	7 (41.18)	2.289	0.130
Feeling of urge, Capacity to verbalize, Hold defecation	6 (33.33)	10 (58.82)		

The values are presented as numbers and percentages (%). ARM, anorectal malformation; TCS, tethered cord syndrome.

## Data Availability

Data supporting reported results are available on request in anonymized manner.
